# Steam activation of waste biomass: highly microporous carbon, optimization of bisphenol A, and diuron adsorption by response surface methodology

**DOI:** 10.1007/s11356-018-3455-3

**Published:** 2018-10-24

**Authors:** Mohamed Zbair, Kaisu Ainassaari, Zouhair El Assal, Satu Ojala, Nadia El Ouahedy, Riitta L. Keiski, Mohammed Bensitel, Rachid Brahmi

**Affiliations:** 1 Laboratory of Catalysis and Corrosion of Materials (LCCM), Department of Chemistry, Faculty of Sciences of El Jadida, University of Chouaïb Doukkali, BP 20, 24000 El Jadida, Morocco; 2 Environmental and Chemical Engineering, Faculty of Technology, University of Oulu, P.O. Box 4300, 90014 Oulu, Finland

**Keywords:** Microporous carbon, Steam activation, Adsorption, Bisphenol A, Diuron, Response surface method

## Abstract

Highly microporous carbons were prepared from argan nut shell (ANS) using steam activation method. The carbons prepared (ANS@H2O-30, ANS@H2O-90, and ANS@H2O-120) were characterized using X-ray diffraction, scanning electron microscopy, Fourier-transform infrared, nitrogen adsorption, total X-ray fluorescence, and temperature-programmed desorption (TPD). The ANS@H2O-120 was found to have a high surface area of 2853 m^2^/g. The adsorption of bisphenol A and diuron on ANS@H2O-120 was investigated. The isotherm data were fitted using Langmuir and Freundlich models. Langmuir isotherm model presented the best fit to the experimental data suggesting micropore filling of ANS@H2O-120. The ANS@H2O-120 adsorbent demonstrated high monolayer adsorption capacity of 1408 and 1087 mg/g for bisphenol A and diuron, respectively. The efficiency of the adsorption was linked to the porous structure and to the availability of the surface adsorption sites on ANS@H2O-120. Response surface method was used to optimize the removal efficiency of bisphenol A and diuron on ANS@H2O-120 from aqueous solution.

**Electronic supplementary material:**

The online version of this article (10.1007/s11356-018-3455-3) contains supplementary material, which is available to authorized users.

**Graphical abstract:**
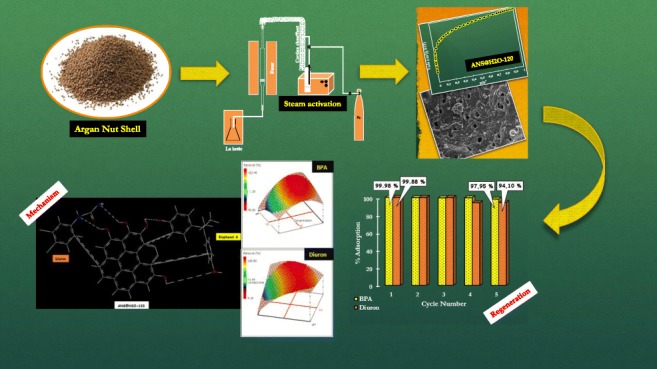
ᅟ

## Introduction

The production of porous carbon materials needed a suitable porous carbon matrix from organic materials. Generally, porous carbon materials are prepared by the chemical or physical procedure. The chemical activation is conventionally carried out simultaneously with the carbonization step in the presence of oxidation catalysts, such as phosphoric acid, sulfuric acid, potassium sulfide, zinc chloride, copper salts, or potassium hydroxide (Fierro et al. 2007; Bulgariu et al. 2011; Sayʇili et al. 2015; Bădescu et al. 2018; Zbair et al. 2018a, b). The raw material is impregnated with one of these chemical agents and then heated under an inert atmosphere between 400 and 700 °C (Sayğılı and Güzel 2018). Physical activation consists of oxidation of the coal at high temperature (700 to 1000 °C) using a weakly oxidizing agent. The gaseous reactants mainly used are air, carbon dioxide, and steam (Kilpimaa et al. 2015; Rezma et al. 2017).

These procedures lead to the development of porous structure in the micropore range (below 2 nm) with a relatively small development of mesopores (2–50 nm). Physical activation process permits to obtain carbons with narrower pore size distributions (Prauchner and Rodríguez-Reinoso 2012). However, chemical activation leads to carbons with a higher fraction of mesopores (Prauchner and Rodríguez-Reinoso 2012). Nevertheless, chemically activated carbons (ACs) generally retain traces of the activating agent responsible for surface oxidation (phosphorus, sulfur, zinc, copper, potassium).

Nowadays, there is a great interest in the development of feasible, inexpensive, high capacity, highly microporous sorbents for toxic metals, organic pollutants, CO_2_ capture, and also can be used as supercapacitor (Ello et al. 2013; Maneerung et al. 2016; Eftekhari 2018; Zbair et al. 2018b). In addition, the pore building of carbon material is the main parameter for adsorption of toxic metals and organic contaminants. AC predominantly consists of micropores (< 2 nm in size); accordingly, adsorption of small molecules, such as low-substituted monoaromatics, can be facilitated by the micropore-filling effect once the molecular size of the adsorbate is close to the pore size of the adsorbent (Ismadji and Bhatia 2001; Nakagawa et al. 2004; Fu et al. 2011). Moreover, steam activation shows various advantages, such as high adsorption capacity, which is mainly attributed to its high surface area and the abundance of hydroxyl and carboxyl groups (-OH and -COOH) (Bouchelta et al. 2008; Maneerung et al. 2016). In addition, Maneerung et al. (2016) found that AC prepared by steam activation has higher surface area (microporous materials) with high adsorption capacity of rhodamine B as compared to those obtained from CO_2_ and N_2_ activations (Maneerung et al. 2016).

Depending on the biomass source and activation conditions, the specific area differs from ∼ 500 to 2000 m^2^/g and the micropore volume from 0.2 to 0.9 cm^3^/g (Mohamed et al. 2010). Among several biomass sources, argan nut shell (ANS) was used in our previous study as raw material to produce AC via chemical activation, and the results show high surface area (1372 m^2^/g) with highest adsorption capacity (1250 mg/g) of bisphenol A (BPA) compared to the literature (Zbair et al. 2018b). Nevertheless, the production of AC from the ANS by physical activation using steam has not been studied. So, it appears to us pertinent to study this activation procedure in more detail, which is more promising to preserve the environment since no chemical reagents are used.

BPA and diuron were selected as model pollutants. This choice was motivated, on the one hand, by their harmful nature for the environment and, on the other hand, by the ease of their dosage in water. In addition, they possess various functional chemical groups.

BPA attracts comprehensive attention because of its extensive use as a monomer in the manufacture of polycarbonates, epoxy resins, and other plastics (Wang et al. 2017a). BPA has been broadly identified in wastewater, groundwater, surface water, and even drinking water (Lane et al. 2015; Lee et al. 2015). Recent studies have shown that it has a potential hormonal activity, which can induce hormonal disruption in an intact organism and as such he is called an “endocrine disruptor” (Bhatnagar and Anastopoulos 2017; Zbair et al. 2018b). However, its environmental impact is poorly documented and the alternatives to BPA are not yet industrialized, which is why this molecule has caught our attention.

On another hand, the herbicide diuron (*N*-(3,4-dichlorophenyl)-*N*,*N*-dimethylurea) is broadly used in cultivation, and its existence in surface and groundwater has consequently augmented (Field et al. 2003; Claver et al. 2006). According to the US Environmental Protection Agency (EPA), diuron was categorized as a “known/likely” carcinogen since 1997 (Liu et al. 2010). Some environmental actions about diuron have been stated, for instance, photolysis (Djebbar et al. 2008), soil degradation (Inoue et al. 2008), and hydrolysis (Feng et al. 2008). Also, the outcomes revealed that diuron was quite stable in water and not sensitive to light. Mainly, the degradation of diuron in soil was very slow. Dores’s investigation (Dores et al. 2009) indicated that diuron was identified in concentration diminishing until 70 days after application in runoff water and soil, totalizing 13.9% through the whole sampling period. Because of its high persistence, groundwater contamination by diuron has become a serious problem (Imache et al. 2008). Adsorption on solid surfaces is an important approach for monitoring the existence of organic contaminants in water. Carbon materials have been suggested as adsorbents to remove hazardous contaminants from polluted waters. ACs are inexpensive materials; they are commonly applied in several fields as adsorbents for toxic metals ions and organic pollutants (Bulgariu et al. 2011; Lin et al. 2013; Anastopoulos et al. 2017, 2018; Zbair et al. 2018a, b).

The goal of this study is then to prepare carbon material from ANS that is abundant Moroccan by-products using steam activation method. The chemical and textural properties of these carbon materials were determined, using different analysis techniques, to highlight their influence on the performance of the carbon in BPA and diuron adsorption. In addition, the response surface method (RSM) was used to get a more systematic view of the adsorption process.

## Experimental section

### Materials and methods

Detailed information on preparation, characterization methods, adsorption experiments, and regeneration protocol is described in the Supporting information.

### Response surface method

The RSM was used to optimize the adsorption of BPA and diuron on ANS@H2O-120. Three main parameters (*X*_1_: pH, *X*_2_: concentration, and *X*_3_: contact time) influencing the adsorption of BPA and diuron onto ANS@H2O-120 were chosen for experimental design (Anfar et al. 2017). For the three parameters studied, the design was elaborated using 2*k* factorial design experiments (*k* = 3), six axial point, and three central points (de Sales et al. 2013). The effects of independent factors on adsorption process were analyzed using a quadratic Eq. (1) as given below: where *Y*, *a*_0_, *a*_i_, *a*_ij_, and *a*_ii_ are the predicted response, constant, linear, interaction, and quadratic coefficients, respectively. *X*_i_ and *X*_j_ are the coded values for the experimental parameters. The levels of the operational parameters and results are given in Table 2S.

1 $$ Y={a}_0+{\sum}_{\mathrm{i}=1}^{\mathrm{k}}{a}_{\mathrm{i}}{X}_{\mathrm{i}}+{\sum}_{\mathrm{i}=1}^{\mathrm{k}}{a}_{\mathrm{i}\mathrm{i}}{X}_{\mathrm{i}}^2+{\sum}_{\mathrm{i}-1}^{\mathrm{k}-1}{\sum}_{\mathrm{j}-1}^{\mathrm{k}}{a}_{\mathrm{i}\mathrm{j}}{X}_{\mathrm{i}}{X}_{\mathrm{j}} $$

## Results and discussion

### Characteristics of prepared carbon materials

**Fig. 1:**
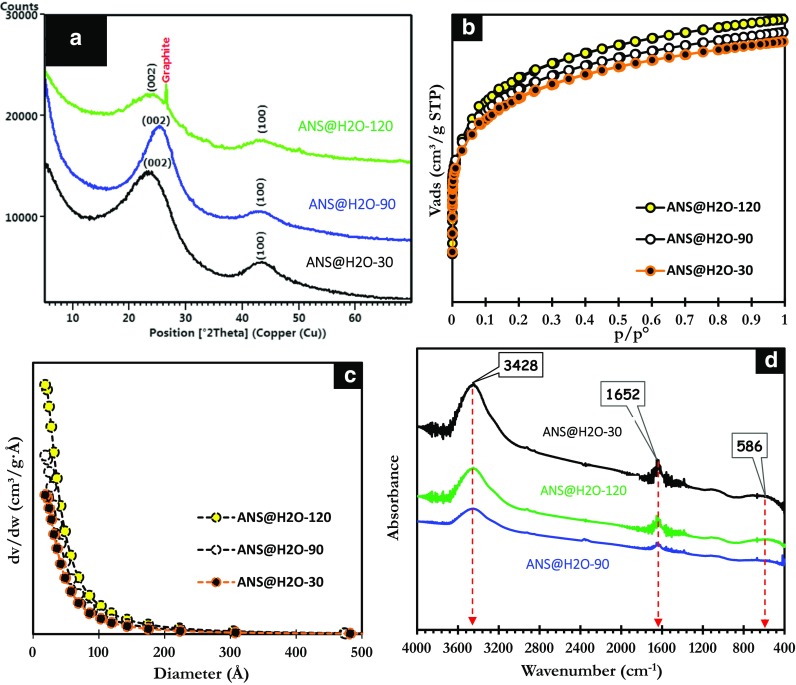
**a** X-ray diffractograms. **b** Adsorption isotherms. **c** Pore size distributions. **d** FTIR spectra of ANS@H2O-30, ANS@H2O-90, and ANS@H2O-120

The diffractograms of ANS@H2O-30, ANS@H2O-90, and ANS@H2O-120 shown in Fig. 1a highlight the presence of broad peaks around 23° and 43°, attributed to Bragg reflections (002) and (100) of amorphous carbon (Hadoun et al. 2013; Wang et al. 2017b). The sharp peak observed at 26.3° for ANS@H2O-120 is assigned to the graphitic plane (002) in agreement with JCPDS 00-75-2078. This result is very interesting, and it clearly shows that for thermal treatments greater than or equal to 2 h, the formation of graphite carbon is favored and its proportion would probably increase with the increase of the duration of the treatment or increase of the temperature (Sivachidambaram et al. 2017; Wang et al. 2017b).

The adsorption isotherms of ANS@H2O-30, ANS@H2O-90, and ANS@H2O-120 shown in Fig. 1b, c are all type I, reflecting their microporous characters (Sing et al. 1985). The greatest specific surface area is obtained for the sample ANS@H2O-120 (2853 m^2^/g). Moreover, it is clearly established that the specific surface area and the pore volume increase with the duration of the heat treatment (Table 1). Indeed, a longer residence time allows the steam to react with the entire surface better and improves the creation and development of new pores, resulting in an increase in specific surface area and pore volume.

**Table 1:** Textural properties of microporous carbons

Microporous carbons	BET surface area (m^2^/g)	Langmuir surface area (m^2^/g)	Total pore volume (cm^3^/g)	Average pore diameter (nm)
ANS@H2O-30	1417	1364	1.56	1.85
ANS@H2O-90	1838	1775	2.01	1.84
ANS@H2O-120	2853	2100	2.83	1.70

Infrared spectroscopy was used for the identification of functional groups present on the surface of the ANS@H2O-30, ANS@H2O-90, and ANS@H2O-120. These groups are often responsible for the adsorbent-adsorbate bonds (Ricordel et al. 2001). The FTIR spectra shown in Fig. 1d reveal the presence of the absorption bands located at 3428 cm^−1^ corresponding to the hydrogen stretching vibrations of the hydroxyl groups O-H. The spectra also show the presence of the band at 1652 cm^−1^ of the C=C bond stretching vibrations of the aromatic rings (Bouchelta et al. 2008; Zbair et al. 2018b) The FTIR band at 586 cm^−1^ is attributed to the deformation of aromatic C-H bonds (Ji et al. 2007).

**Fig. 2:**
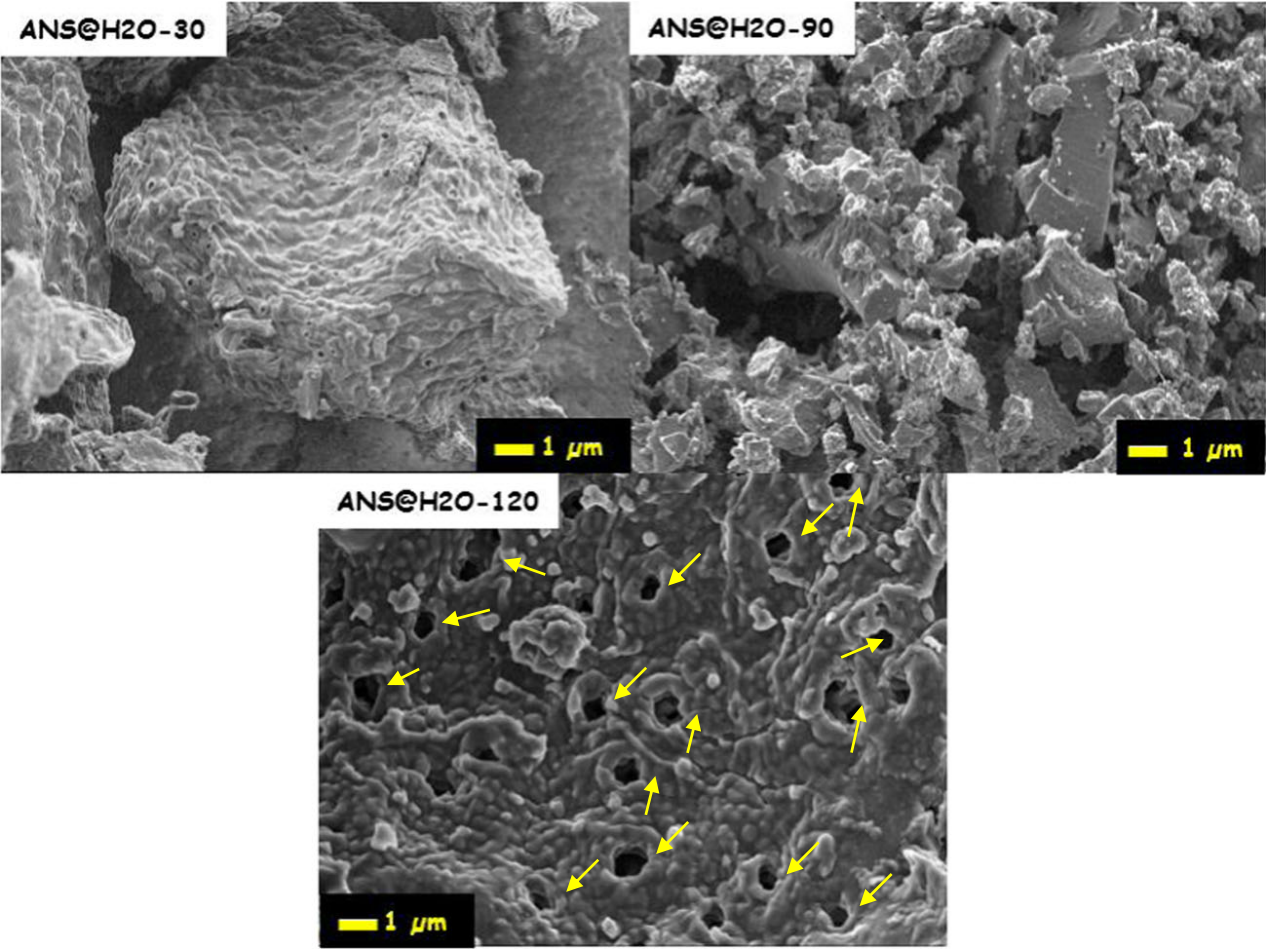
SEM images of samples ANS@H2O-30, ANS@H2O-90, and ANS@H2O-120

The surface analysis of ANS@H2O-30, ANS@H2O-90, and ANS@H2O-120 by scanning electron microscope (Fig. 2) shows the evolution of morphology with the activation time of the heat treatment. Thus, the surface of the ANS@H2O-120 develops a more developed porous structure than those of the ANS@H2O-30 and ANS@H2O-90 samples, which is in perfect agreement with its larger surface area. The textural properties of ANS@H2O-120 are favorable for good adsorption performance of organic pollutants.

**Fig. 3:**
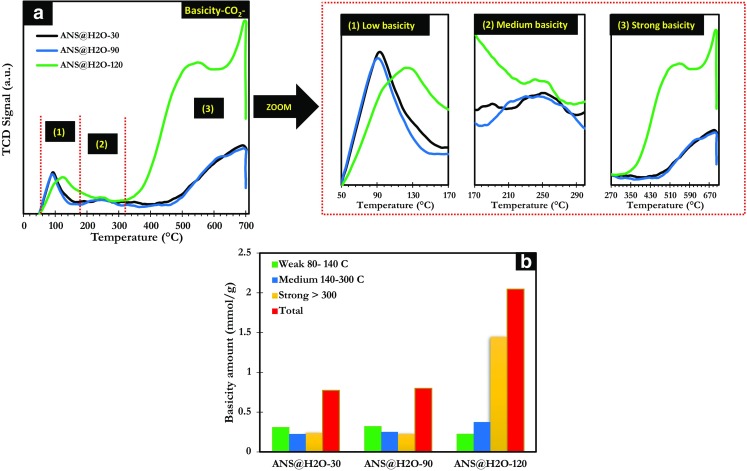
**a** Temperature-programmed desorption (TPD) of CO_2_. **b** Amount of different basic sites

Temperature-programmed desorptions (TPD) of CO_2_ and NH_3_ were performed to determine the amount and the strength of basic and acid sites of ANS@H2O-30, ANS@H2O-90, and ANS@H2O-120. The CO_2_-TPD profiles showed the peaks in the temperature range 50–140, 140–300, and superior to 300 °C are representing desorption of CO_2_ from weak, medium, and strong basic sites (Cho et al. 2005). Generally, the carbonaceous materials obtained from biomass have plenty of hydroxyl groups (-OH), which can react with CO_2_ (Meng et al. 2006). As shown, in Fig. 3a, the profile of all microporous materials shows one peak between 50 to 170 °C, related to the existence of the low amount of weak basic sites. Furthermore, the peaks observed between ∼ 170 to 300 °C for all microporous materials suggest the presence of medium basic sites. The strong basic sites were observed for all the samples, but the ANS@H2O-120 sample exhibited a higher amount of basic sites than the other samples. This result shows that increasing the time of activation from 30 to 120 min influenced in the strength and quantity of the basic sites (Fig. 3b) giving the material improved basic character after 120 min of treatment.

**Fig. 4:**
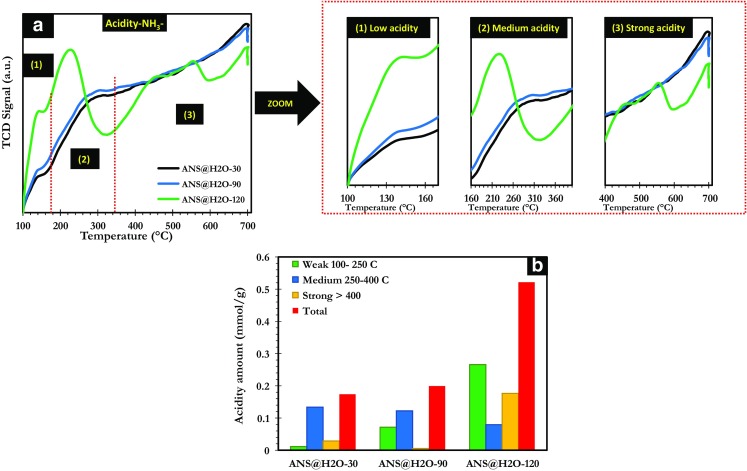
**a** Temperature-programmed desorption (TPD) of NH_3_. **b** Amounts of different acid sites

The NH_3_-TPD profiles of ANS@H2O-30, ANS@H2O-90, and ANS@H2O-120 are shown in Fig. 4a. Low, medium, and strong acid sites are observed in the temperature range of 100–250, 250–400, and superior to 400 °C, respectively (Arena et al. 1998; El Assal et al. 2017). ANS@H2O-30 and ANS@H2O-90 samples were presenting almost similar desorption profiles with only two small peaks. The maxima of the first and second peak were located around 140 and 300 °C. These peaks are corresponding to the presence of weak and medium acid sites. Furthermore, ANS@H2O-90 contained only the very small amount of strong acid sites compared to the other materials (Fig. 4b). The third sample ANS@H2O-120 had weak, medium, and strong acid sites presented by the peaks with maxima observed in the interval of temperature between 100 and 250, 250 and 400, and superior to 400 °C, respectively. The latter sample is presenting more acidic sites followed by ANS@H2O-90 and finally ANS@H2O-30, which is presenting the lowest amount of acid sites. The CO_2_ and NH_3_ TPD analyses demonstrate that the time of activation treatment is influencing both: strength and quantity of acidity and basicity.

The elemental compositions of ANS@H2O-30, ANS@H2O-90, and ANS@H2O-120 were analyzed with X-ray fluorescence and the results obtained are collected in Table 2. These results highlight the presence of a large number of elements with high concentrations in the ANS. The physical activation leads to a decrease and even to a disappearance of certain elements, in particular when increasing the time of the physical activation during the preparation. Taking Al as an example, the concentration was 142 mg/kg and after 30 min of steam activation was decreased to 92 mg/kg, then to 77 mg/kg (90 min of activation) and finally at 120 min the Al was disappeared totally, which may be attributed to the thermal treatment during the preparation.

**Table 2:** Elemental composition of ANS, ANS@H2O-30, ANS@H2O-90, and ANS@H2O-120

Elements	ANS (mg/kg)	ANS@H2O-30 (mg/kg)	ANS@H2O-90 (mg/kg)	ANS@H2O-120 (mg/kg)
Al	142	92	77	–
Mg	214	–	–	–
P	562	398	320	141
S	288	120	21	9
Cl	663	451	98	9
K	1771	954	168	162
Na	25,427	16,950	14,990	14,530
Ca	1524	314	250	58
Mn	6	3	2	0.78
Fe	186	47	35	23
Cu	2	0.9	0.7	0.24
Zn	7	6	2	2
As	0.43	0.2	–	–
Br	2	1	1	0.5

### BPA and diuron removal by microporous carbons

**Fig. 5:**
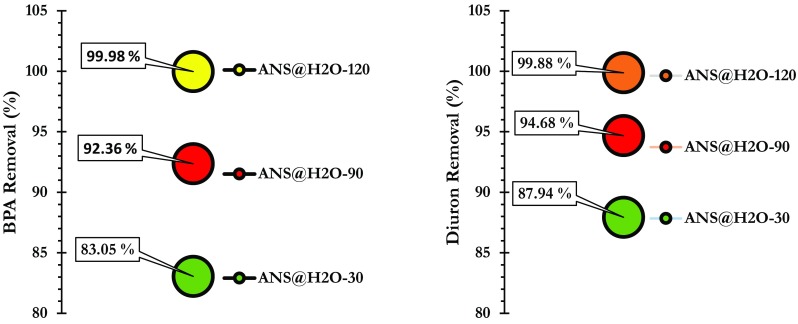
Results of the preliminary adsorption of BPA and diuron by ANS@H20-30, ANS@H20-90, and ANS@H20-120 (BPA: *C*_0_ = 60 mg/L; diuron: *C*_0_ = 40 mg/L; *m* = 0.01 g; agitation speed = 200 rpm)

Preliminary adsorption tests were carried out to find out the BPA and diuron removal proficiencies onto ANS@H2O-30, ANS@H2O-90, and ANS@H2O-120. From Fig. 5, we conclude that ANS@H2O-120 had a higher adsorption efficiency for BPA and diuron than ANS@H2O-30 and ANS@H2O-90. This can be explained by the higher specific surface area of ANS@H2O-120 compared to that of ANS@H2O-30 and ANS@H2O-90 (Table 1). Hereafter, the further adsorption experiments were performed to evaluate and investigate the BPA and diuron adsorption characteristics on ANS@H2O-120 only.

### Effect of pH

**Fig. 6:**
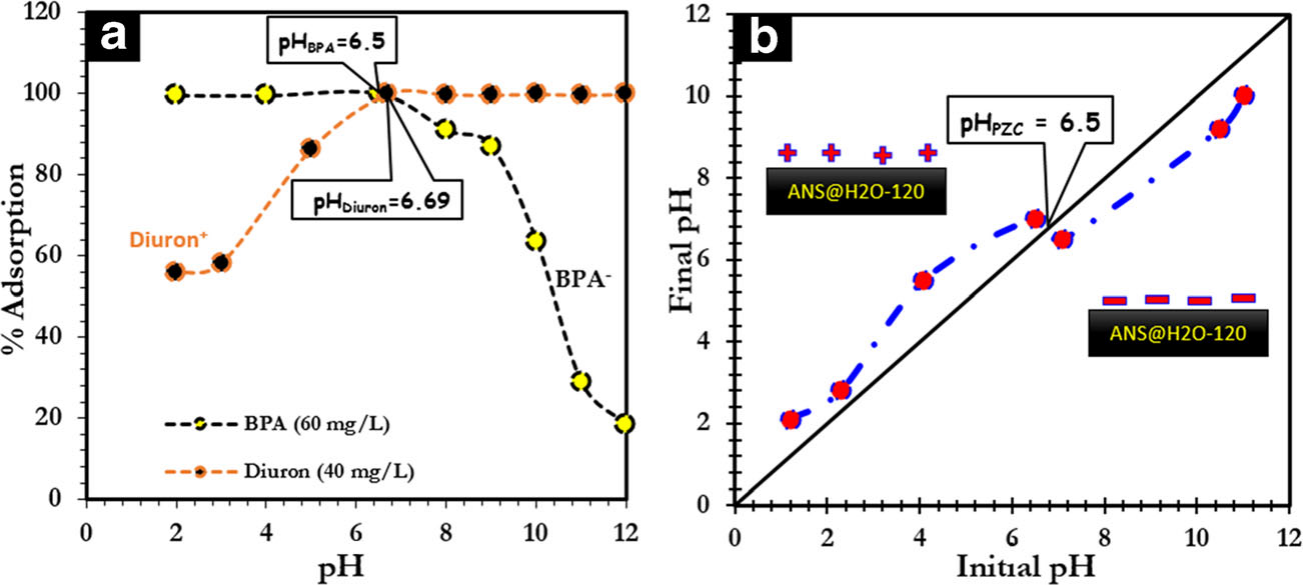
**a** Effect of pH on adsorption of BPA and diuron. **b** Determination of the point of zero charge for ANS@H20-120

The effect of pH of the solution on the adsorption performance of ANS@H2O-120 was studied by varying the pH between 2.0 and 12.0. It was observed that the adsorption of BPA on ANS@H2O-120 reaches a maximum in acidic medium, up to pH = 6.5, corresponding to a yield close to 99% (Fig. 6a). Above 6.5, the adsorption efficiency decreases with increasing pH. Indeed, the surface of ANS@H2O-120 has a net positive charge for pH lower than its pH_PZC_ (6.5) and a net negative charge beyond pH_PZC_ (Fig. 6b), whereas BPA is under its neutral form for a pH lower than 8 (Bautista-Toledo et al. 2005) and deprotonation (mono-anion of bisphenolate) becomes significant around pH = 8 (Sui et al. 2011). As a result, the decrease in adsorption efficiency observed at very high pH values results from repulsive electrostatic interactions between the negatively charged ANS@H2O-120 surface and the bisphenolate anion (Bautista-Toledo et al. 2005; Tsai et al. 2006). In the case of diuron, the percentage of adsorption ANS@H2O-120 (Fig. 6a) remains stable between pH = 6.69 and pH = 12.0 corresponding to a yield of 99% and decreases below pH = 6.69. Indeed, diuron acts as a cationic species at pH < 6.69 and as a neutral molecule with a pH greater than 6 (Fontecha-Cámara et al. 2007). On the other hand, the surface of ANS@H2O-120 is positively charged for a pH lower than the pH_PZC_. Therefore, for a lower pH, repulsive electrostatic interactions between the surface of ANS@H2O-120 and diuron, both positively charged, decrease the efficiency of adsorption. At a pH = 6.69, the diuron molecule and the surface of ANS@H2O-120 are both neutral, which favors non-electrostatic interactions, leading to increased adsorption efficiency. At higher pH, the diuron molecule is neutral while the surface of ANS@H2O-120 is negative, which does not favor electrostatic interactions and therefore maintains the relatively constant diuron adsorption rate (Fontecha-Cámara et al. 2007).

### Adsorption kinetics

The adsorption kinetic studies of BPA and diuron on ANS@H2O-120 were carried out by varying the contact time from 0 to 180 min. Approximately, 10 mg of ANS@H2O-120 was added to 100 mL of aqueous solution (i.e., BPA or diuron) with an initial concentration of 60 mg/L for BPA and 40 mg/L for diuron. Then, the mixture was stirred at room temperature.

**Fig. 7:**
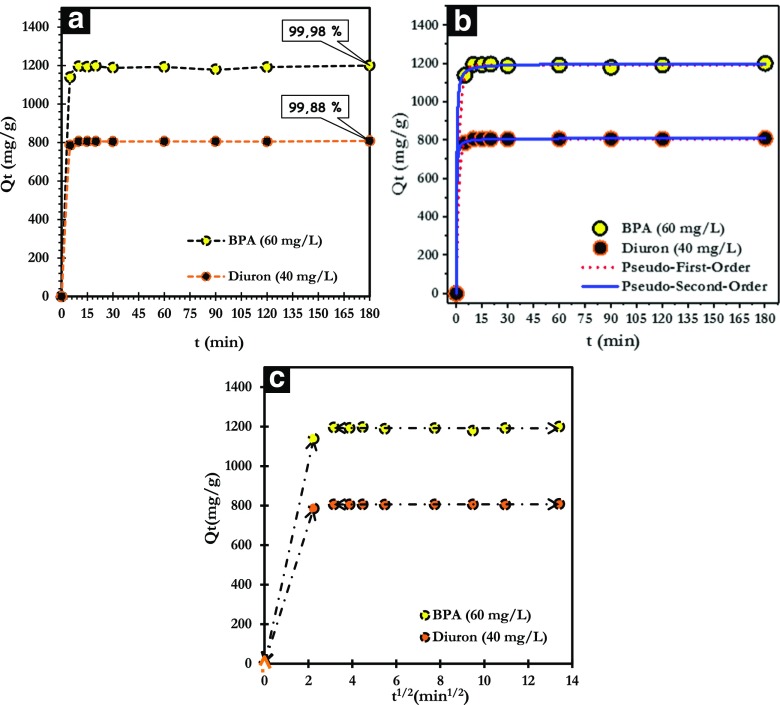
**a** Effect of contact time. **b** Pseudo-first-order and pseudo-second-order-models. **c** Intraparticle diffusion model

The outcomes obtained (Fig. 7a) display that the adsorption rate is very fast and that the adsorption reaches the maximum after 15 min of solid/liquid contact, reaching the adsorption of 1200 mg/g corresponding to 99.98% removal of BPA and 809 mg/g, equivalent to 99.88% removal of diuron.

The experimental results of BPA and diuron on ANS@H2O-120 were fitted using nonlinear pseudo-first-order and pseudo-second-order models (Fig. 7b). The kinetic parameters are gathered in Table 3. The best kinetic model established for the adsorption is chosen according to the value of the correlation coefficient (*R*^2^), which must be as close as possible to 1. According to Table 3, the both models have correlation coefficients close to the unity (*R*^2^ = 0.999) for both adsorbates. However, the quantities of molecules (BPA and Diuron) adsorbed on ANS@H2O-120 at equilibrium are very close to those calculated by the pseudo-second-order model compared to pseudo-first-order model. This indicates that the pseudo-second-order model describes best the kinetics of adsorption of BPA and diuron on ANS@H2O-120.

**Table 3:** Kinetic adsorption parameters of BPA and diuron on ANS@H2O-120

	*Q*_e,exp_ (mg/g)	Pseudo-first-order			Pseudo-second-order		
		*Q*_e,cal_ (mg/g)	*K*_1_ (1/min)	*R* ^2^	*Q*_e,cal_ (mg/g)	*K*_2_ (g/mg min)	*R* ^2^
BPA	1200	1192	0.62	0.999	1199	0.004	0.999
Diuron	809	806	0.73	0.999	809	0.010	0.999

The intraparticle diffusion model was used to describe the mechanism of BPA and diuron transfer to the surface of ANS@H2O-120. As it can be seen from Fig. 7c, the intraparticle diffusion model shows that the adsorption process takes place in two stages (Weber and Morris 1963):

(i) The first step is the diffusion of the BPA and diuron into the outer surface of ANS@H2O-120;
(ii) The second stage is attributed to intraparticle diffusion and adsorption equilibrium (AL-Othman et al. 2012; Suresh et al. 2011).

### Adsorption isotherms and thermodynamics

**Fig. 8:**
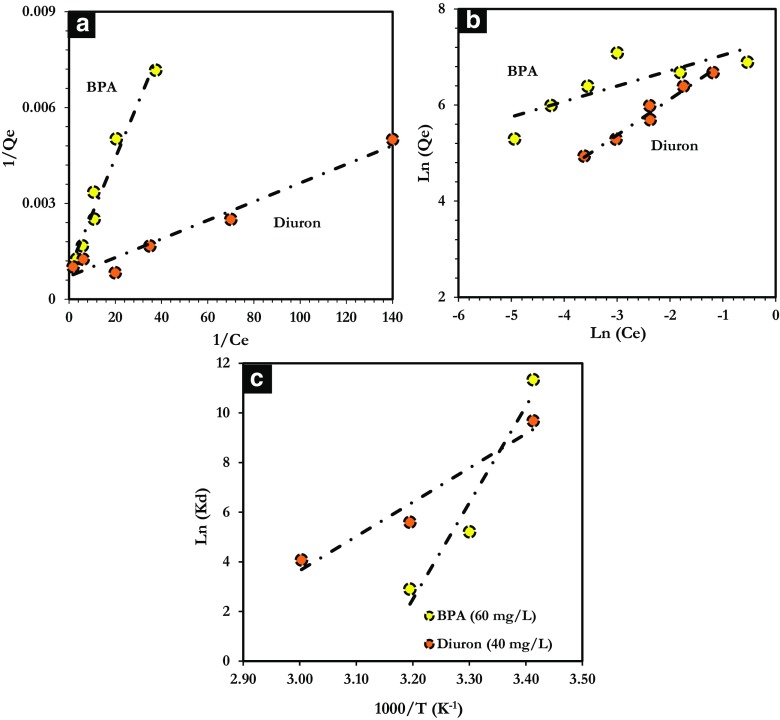
Linear modeling by **a** Langmuir model, **b** Freundlich model, **c** Plot of *Ln*(*Kd*) vs. 1000/*T* for thermodynamic parameters

The Langmuir and Freundlich models were applied to the experimental data obtained from adsorption of BPA and diuron on ANS@H2O-120 (Fig. 8a, b). The adsorption parameters at equilibrium are collated in Table 4. From these results, the Langmuir model describes better the adsorption of BPA and diuron on ANS@H2O-120, as shown by the coefficients of correlation is close to unity (${R}_{\mathrm{BPA}}^2$ = 0.9576 and ${R}_{\mathrm{BPA}}^2$ = 0.9646) compared to those obtained with the Freundlich model (${R}_{\mathrm{BPA}}^2$ = 0.6192 and ${R}_{\mathrm{BPA}}^2$ = 0.6228). This reflects mainly to a micropore filling of BPA and diuron on the microporosity of ANS@H2O-120. Additionally, the length of BPA and diuron molecules is 0.94 and 0.75 nm, respectively, and the average pore size of ANS@H2O-120 is 1.7 nm, which indicate that BPA and diuron molecules can enter easily to the porous structure of ANS@H2O-120. Besides, the separation factor (*R*_L_) is between 0 and 1, which proves that the adsorption of BPA and diuron on ANS@H2O-120 is favorable. Clearly, the outcomes obtained, in terms of adsorption capacity, are higher than most outcomes obtained on other types of adsorbents, stated in the literature (for example, AC, graphene, and graphite) (Table 4).

**Table 4:** Langmuir and Freundlich parameters and compared maximum adsorption capacity of BPA and diuron on ANS@H2O-120 with different adsorbents

	Langmuir				Freundlich		
	*Q*_max_ (mg/g)	*K*_L_ (L/mg)	*R*_L_	*R* ^2^	*K*_F_ (mg/g)/(mg/L)^n^	*n*	*R* ^2^
BPA	1408	24.483	0.001	0.9576	1569	0.757	0.6192
Diuron	1087	5.412	0.005	0.9646	1166	1.343	0.6228
Microporous carbon		Specific surface area (m^2^/g)	Adsorption capacity (mg/g)		Reference		
Bisphenol A							
ANS@H2O-120		2853	1408		Current study		
Activated carbon (AC-HP)		1372	1250		(Zbair et al. 2018b)		
Activated carbon (Takeda)		1119	23.5		(Nakanishi et al. 2002)		
Graphene		327	181.6		(Xu et al. 2012)		
mesoporous carbon (CMK3)		920	296		(Sui et al. 2011)		
Activated carbon		1760	432.3		(Liu et al. 2009)		
Activated carbon powder		–	771.2		(Li et al. 2014)		
CoFe_2_O_4_/activated carbon		–	727.2		(Li et al. 2014)		
Diuron							
ANS@H2O-120		2853	1087		Current study		
Activated carbon		2128	292.9		(López-Ramón et al. 2007)		
Activated carbon fiber		1709	667.2		(López-Ramón et al. 2007)		
Activated carbon		776	279.4		(Ángeles Fontecha-Cámara et al. 2006)		
Activated carbon		1139	51.2		(Bahri et al. 2012)		
Carbon nanotubes		258.6	39.6		(Sun et al. 2012)		

Information on the effect of temperature on the adsorption process on ANS@H2O-120 was achieved by varying the solution temperature from 293 to 333 K while keeping the other parameters constant. The results obtained are used to determine thermodynamic parameters (Δ*H*°, Δ*S*°, and Δ*G*°) related to the adsorption reaction of BPA and diuron on ANS@H2O-120. The variations of *Ln*(*Kd*) as a function of (1000/*T*) are shown in Fig. 8c and the thermodynamic parameters are grouped in Table 5. The negative standard enthalpy (Δ*H*° < 0) shows the exothermic nature of the adsorption of the two studied pollutants. The values of standard free enthalpy at different temperatures are negative (Δ*G*° < 0) revealing a spontaneous adsorption phenomenon. The negative entropy (Δ*S*°) of BPA and diuron adsorption indicates that randomness is decreased at the solid-liquid interface (Badruddoza et al. 2011). Moreover, the adsorption process is supposed to be physisorption, when the values of Δ*G*° are between 0 and − 20 kJ/mol, whereas for chemisorption, the values of Δ*G*° are generally between − 80 and − 400 kJ/mol (Khan et al. 2017; Zbair et al. 2018b). In this study, Δ*G*° obtained ranged between − 7 to − 17 kJ/mol for BPA and − 10 to − 18 kJ/mol for diuron, which means that the adsorption process of BPA and diuron on ANS@H2O-120 is physisorption.

**Table 5:** Thermodynamic parameters of BPA and diuron adsorption on ANS@H2O-120

Thermodynamics	Δ*H*° (J/mol)	Δ*S*° (J/mol K)	Δ*G*° (kJ/mol)		
parameters			293 K	303 K	313 K
BPA	− 323.02	− 1013	− 17.620	− 13.124	− 7.557
Diuron	− 114.6	− 313.4	− 18.580	− 14.104	− 10.610

### Regeneration and reuse

**Fig. 9:**
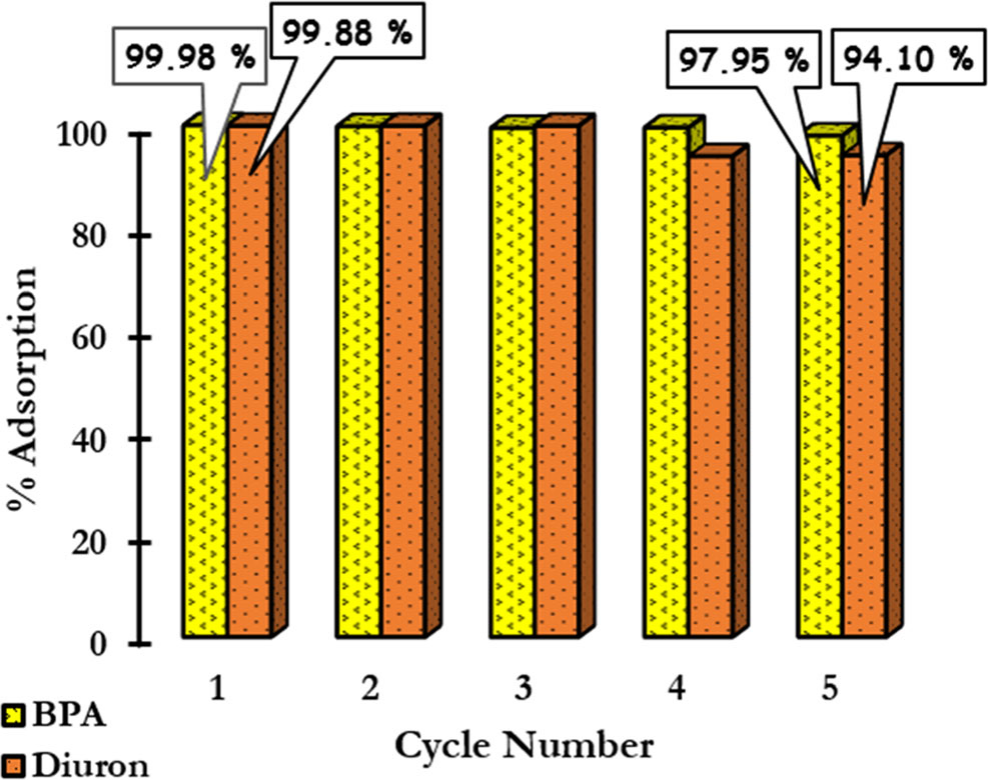
Regeneration and reuse of ANS@H2O-120 using ethanol

The regeneration of ANS@H2O-120 was carried out using ethanol to desorb the adsorbed pollutants (BPA and diuron). The adsorption efficiency of BPA and diuron on regenerated ANS@H2O-120 is shown in Fig. 9. The results show that the efficiency of the adsorption on ANS@H2O-120 remains practically constant (decrease in the order of 2.03%) after 5 cycles of adsorption/desorption of BPA. In the case of diuron after 5 adsorption-desorption cycles, the adsorption efficiency decreases in the order of 5.78%. Figure 1S shows the XRD patterns of fresh ANS@H2O-120 and the recycled ANS@H2O-120 after adsorption of BPA and diuron (BPA/ANS@H2O-120 and diuron/ANS@H2O-120). The results show that there is no modification occurred on the phase structure of recycled adsorbents, which indicate that our regeneration protocol using ethanol is promising for regeneration studies.

In general, the experimental results obtained suggest that ANS@H2O-120 prepared by steam activation can be used as a recyclable, separable, and effective adsorbent for the decontamination of wastewater.

### Adsorption mechanism

**Fig. 10:**
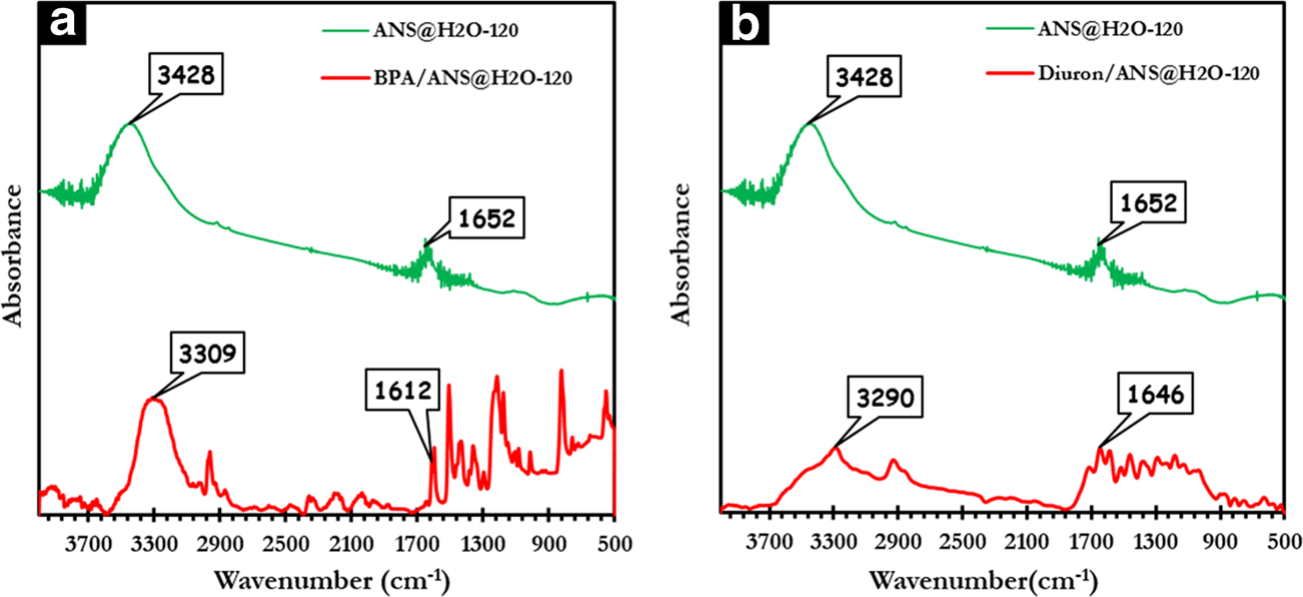
FTIR spectra of ANS@H2O-120 before and BPA and diuron adsorption

In order to elucidate the adsorption mechanism of BPA and diuron on ANS@H2O-120, FTIR spectroscopy was used to study the ANS@H2O-120 before and after adsorption (Fig. 10). In general, the possible mechanisms for the adsorption of organic pollutants on carbonaceous materials are an electrostatic attraction, hydrogen bond formation, *n*-*π* interaction, *π*-*π* interaction, and pore filling (Coughlin and Ezra 1968; Tran et al. 2017a).

**Fig. 11:**
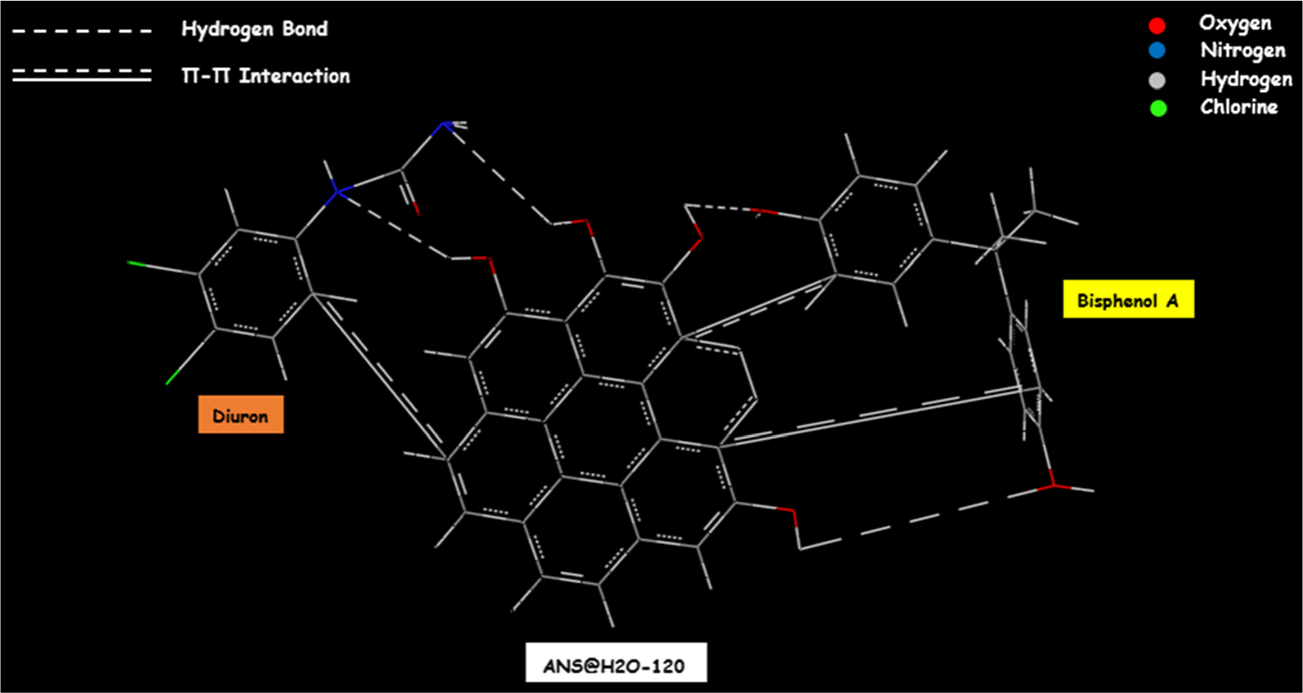
Proposed adsorption mechanism (except pore filling)

The FTIR spectra of BPA loaded ANS@H2O-120 show that the peaks around 3428 and 1652 cm^−1^ are slightly shifted from their original position to 3309 and 1612 cm^−1^, respectively (Fig. 10a). In the same case for diuron, the peaks around 3428 and 1652 cm^−1^ are slightly shifted from their original position to 3290 and 1646 cm^−1^, respectively (Fig. 10b). Knowing that the peak located at 3428 cm^−1^ is assigned to -OH groups and the peak observed at 1652 cm^−1^ is corresponding to C=C of the aromatic rings, the shift can be interpreted by the formation of hydrogen bonds between hydroxyl groups present in both BPA molecule and ANS@H2O-120 and hydrogen bonds between -OH present in ANS@H2O-120 with nitrogen atom present in the diuron molecule (Coughlin and Ezra 1968; Tran et al. 2017a, b). The schematic representation of the *π*-*π* interaction and hydrogen bonding between BPA and diuron on ANS@H2O-120 is presented in Fig. 11.

### Response surface method

In this study, the parameters for maximum BPA and diuron adsorption onto ANS@H2O-120, such as pH, concentration, and contact time, were optimized using central composite design (CCD) under RSM (Anfar et al. 2017). The experimental results presented in Table 2S were used to develop the polynomial equation below (Eqs. (2) and (3)), which was used to optimize the parameters for adsorption of BPA and diuron on ANS@H2O-120. The results of ANOVA for BPA and diuron adsorption on ANS@H2O-120 showed *P* value less than 0.05 in both models. Therefore, both developed models explain very well the phenomenon studied, with a confidence level of 95% (Table 3S). According to the ANOVA, the term “*P* value” shows the degree of effectiveness of each model (*P* value < 0.05 is considered effective and those with *P* values > 0.05 are regarded as ineffective) (Ahsaine et al. 2018).

2 $$ R\%\left(\mathrm{BPA}\right)=98.928\hbox{--} 9.749{X}_{\mathrm{pH}}\hbox{--} 0.949{X}_{\mathrm{C}}+1.387{X}_{\mathrm{C}\mathrm{T}}\hbox{--} 7.688{X^2}_{\mathrm{pH}}\hbox{--} 1.982{X^2}_{\mathrm{C}}\hbox{--} 1.681{X^2}_{\mathrm{C}\mathrm{T}}+3.809{X^2}_{\mathrm{pH}-\mathrm{C}}+2.594{X^2}_{\mathrm{pH}-\mathrm{C}\mathrm{T}}+1.594{X^2}_{\mathrm{C}-\mathrm{C}\mathrm{T}} $$

3 $$ R\%\left(\mathrm{diuron}\right)=98.56+12.432\ {X}_{\mathrm{pH}}\hbox{--} 0.255\ {X}_{\mathrm{C}}+5.722\ {X}_{\mathrm{C}\mathrm{T}}\hbox{--} 10.012\ {X^2}_{\mathrm{pH}}+0.270\ {X^2}_{\mathrm{C}}\hbox{--} 0.031\ {X^2}_{\mathrm{C}\mathrm{T}}+0.223\ {X^2}_{\mathrm{pH}-\mathrm{C}}\hbox{--} 9.287\ {X^2}_{\mathrm{pH}-\mathrm{C}\mathrm{T}}\hbox{--} 1.277\ {X^2}_{\mathrm{C}-\mathrm{C}\mathrm{T}} $$

**Fig. 12:**
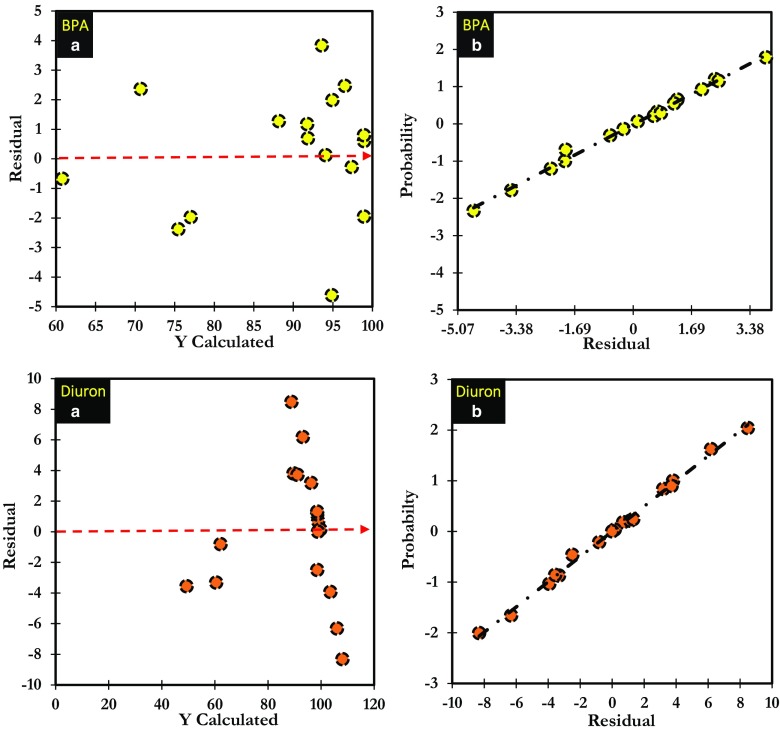
Normality test: **a** plot of raw residuals vs Y_calculated_, **b** probability as a function of residual

As shown in Table 3S, the *R*^2^ of BPA and diuron models was found to be 0.964 and 0.937, respectively. According to the value of these coefficients, we can conclude that there is a good agreement between the experimental and predicted responses of BPA and diuron removal percentage on ANS@H2O-120. From all coefficients presented in Table 3S, we found that only “*X*^2^_pH_” and “*X*^2^_pH-C_” interactions affect the BPA adsorption significantly and “*X*^2^_pH_” and “*X*^2^_pH-CT_” in the case of diuron. In the same context, as shown in Fig. 12, the homogenous distribution of the residues on the “0” axis and the absence of the Henry line confirm the normality of the residues (Tanyildizi 2011).

**Fig. 13:**
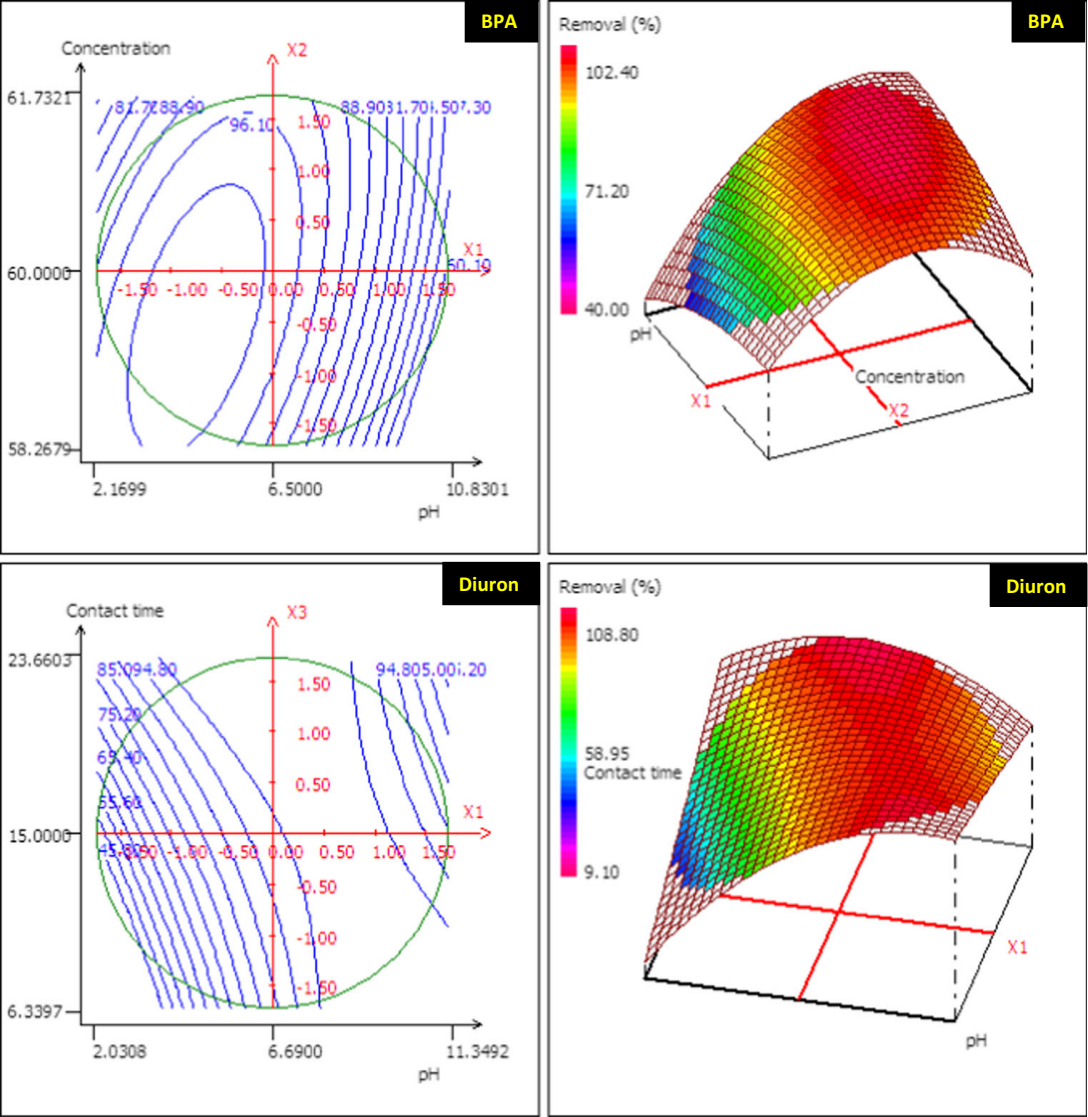
RSM and corresponding contour plot of BPA and diuron

Based on RSM and the contour plot (Fig. 13), we are able to optimize the adsorption process of BPA and diuron on ANS@H2O-120. The optimum parameters obtained from RSM and contour plot are as follows:

- BPA: pH = 6.5, 15 min, and 60 mg/L.
- Diuron: pH = 6.69, 15 min, and 40 mg/L.

Under these conditions, we found experimentally the removal of 99.24% for BPA and 99.46% for diuron adsorption, while the predicted values by the model were as follows: 98.85% for BPA adsorption and 99.53% for diuron adsorption. This shows that the model follows rather well the experimentally determined values of adsorption.

## Conclusion

In this study, different microporous carbon materials derived from ANS were used effectively for the adsorptive removal of BPA and diuron from aqueous solution. From the prepared carbons, the one activated during 120 min represented the best initial characteristics due to the high surface area and well-developed porosity. Therefore, it was selected for the adsorption tests. The adsorption process on ANS@H2O-120 was described well using pseudo-second-order kinetic and Langmuir isotherm (micropore filling) models with maximum adsorption capacities of 1408 mg/g for BPA and 1087 mg/g for diuron. In addition, the values of thermodynamic parameters indicated that the adsorption process on ANS@H2O-120 was spontaneous and exothermic in nature. The regeneration of ANS@H2O-120 by ethanol was successful even after five times of adsorption-regeneration cycles (a very low decrease from 99.98 to 97.95% for BPA and from 99.88 to 94.10% in the case of diuron was observed). RSM was used to model the adsorption process to find out the optimal parameters for the process. It was found that the developed model predicts well the results, and the optimal parameter values for the BPA adsorption were as follows: pH = 6.5, 15 min, and 60 mg/L and for diuron: pH = 6.69, 15 min, and 40 mg/L.

## Electronic supplementary material

ESM 1 (DOCX 68.8 kb)
