# Supplementary material for: Steam activation of waste biomass: highly microporous carbon, optimization of bisphenol A, and diuron adsorption by response surface methodology
Source: Environ Sci Pollut Res Int. 2018 Oct 24;25(35):35657–71. doi: 10.1007/s11356-018-3455-3 (PMC6280859; doi:10.1007/s11356-018-3455-3)
Supplement: Supplementary file 1 — (DOCX 68.8 kb) [file 11356_2018_3455_MOESM1_ESM.docx]

**Steam Activation of Waste Biomass: Highly Microporous Carbon, Optimization of Bisphenol A and Diuron Adsorption by Response Surface Methodology**

**Mohamed Zbair^1,2,*^, Kaisu Ainassaari^2^ , Zouhair El Assal^2^, Satu Ojala^2^, Nadia El Ouahedy^1^, Riitta L. Keiski ^2^, Mohammed Bensitel^1^, Rachid Brahmi^1^**

^1^ Laboratory of Catalysis and Corrosion of Materials (LCCM), Department of Chemistry, Faculty of Sciences of El Jadida, University of Chouaïb Doukkali, BP 20, 24000 El Jadida, Morocco

^2^ Environmental and Chemical Engineering, Faculty of Technology, University of Oulu, P.O. Box 4300, 90014 Oulu, Finland

**Supporting Information**

To whom correspondence must be addressed: [zbair.mohamed@gmail.com](mailto:zbair.mohamed@gmail.com); Tel:+358 465 232 839

**Material**

All chemicals were of analytical reagent grade and used without further purification: Sodium hydroxide NaOH (purity 98%), Hydrochloric acid (purity > 37%), Bisphenol A and diuron (purity 99%) were purchased from Sigma-Aldrich.

**Preparation of microporous carbon**

The preparation process consists of the carbonization of argan nut shell (ANS) under nitrogen atmosphere followed by steam activation. The carbonization of argan nut shell (ANS) was performed under a continuous flow of purified nitrogen gas with a flow rate of 50 mL/min. The precursor was heated from room temperature to 500 °C (5 °C/min) and maintained at this final temperature for 60 min. After the carbonization step, a mass of obtained material was placed in the quartz reactor. The assembly was then brought to the furnace where the used temperature program was as follows:

- A rise of 10 °C/min from room temperature to 800 ° C;
- A 30 minutes, 90 minutes or 120-minutes stage at the highest temperature was used to verify the influence of the heat treatment duration on the properties of the carbon materials.
- Finally, the oven was let to cool freely until the ambient temperature. The activation was carried out under nitrogen and steam with a total flow rate of 50 mL/min, maintained throughout the duration of the experiment.

Three carbon materials were prepared using steam activation method by varying the duration time at 800 °C. The prepared carbon materials were labeled as: ANS@H2O-30, ANS@H2O-90, and ANS@H2O-120.

**Adsorption experiments**

The effect of the initial pH was studied by mixing 10 mg of ANS@H2O-120 and 200 mL of BPA (60 mg/L) and for diuron 40 mg/L in water, the initial pH was adjusted by 0.1 mol/L HCl and NaOH solutions. Kinetic studies were done with a fixed amount 10 mg of ANS@H2O-120 and initial BPA concentration 60 mg/L and 40 mg/L for diuron. The volume of 200 mL BPA and diuron solutions was agitated (200 rpm) at different temperatures (293, 313, and 333 K) for 180 min and the solution was filtered to remove the ANS@H2O-120 adsorbent. Residual BPA and diuron concentrations were determined by spectrophotometry at the maximum absorbance wavelength (λ_max_) of 274 nm for BPA and 248 nm for diuron. The isotherm studies were carried out by agitating 200 mL solution of BPA of desired concentrations (5-60 mg/L) and for diuron between 5-40 mg/L mixed with 10 mg of ANS@H2O-120 for 180 min. Table 1S highlights the mathematical models using in this study to fit the data.

**Regeneration protocol**

The regeneration of the ANS@H2O-120 after BPA and diuron adsorption was carried out by mixing BPA-loaded ANS@H2O-120 or diuron loaded ANS@H2O-120 with 20 mL of ethanol for 4 h and then filtered and dried at 80 °C. Five cycles of BPA and diuron adsorption/desorption studies were carried out accordingly.

**Characterization methods**

XRD patterns were collected using a Bruker eco D8 Advance diffractometer operating at 45 kV/35mA, using CuKa radiation with Ni filter, and working in continuous mode with a step size of 0,02. Data were collected over a range of 05-70°. The N2 adsorption-desorption isotherms of ANS@H2O-30, ANS@H2O-90, and ANS@H2O-120 were measured using a Micromeritics ASAP 2020 instrument to determine surface area, pore volume, and pore size distribution. The morphological characteristics of ANS@H2O-30, ANS@H2O-90, and ANS@H2O-120 were analyzed using a Field Emission Scanning Electron Microscopy ZEISS ULTRA plus. The acidity and basicity measurements of ANS@H2O-30, ANS@H2O-90, and ANS@H2O-120 were done using temperature programmed desorption of NH_3_ and CO_2_ by an AutoChem II 2920 device. Prior to NH_3_-TPD analysis, the sample (about 100 mg) was pre-treated with Helium (He) at 700 °C for 30 min. Then the sample was cooled to 100 °C, followed by adsorption of 15% NH_3_ in He (at 100°C) for 60 min and flushing with He was made for 30 min, in order to remove the physisorbed NH_3_. The NH_3_ desorption was carried out from 100 to 700 °C, and left for 10 min at this temperature. The flow rate used was 50 cm^3^/min and temperature rise was 10 °C/min during the NH_3_-TPD analysis. After NH_3_-TPD analysis, the sample was cooled to room temperature (RT) prior CO_2_-TPD. The sample was flushed with H_2_ (30 cm^3^/min) from RT to 500 °C with 10°C/min for 30 min, then cooled to 50 °C and flushed with Ar with 50 cm^3^/min for 5 min. The adsorption of 5%CO_2_/Ar (50 cm^3^/min) was done at 50 °C for 60 min then the physisorbed CO_2_ was flushed by Ar (50 cm^3^/min) for 60 min. The thermodesorption of CO_2_ done by Ar (50 cm^3^/min) from 50 to 700 °C and left for 10 min at this temperature. The concentration of desorbed NH_3_ and CO_2_ was analysed by a TCD detector and the total acidity and basicity of samples was determined by integration of the area between 100-700 °C and 40-700 °C. The quantification of element concentrations in the argan nut shell (ANS), ANS@H2O-30, ANS@H2O-90, and ANS@H2O-120 was done by using an S2 PICOFOX TXRF (Bruker AXS Microanalysis). Before analysis, around 100 mg of sample was grounded in a mortar and then suspended in a detergent solution of standard (Se) with concentration of 30 mg/L. The PZC (point of zero charge) of ANS@H2O-120 was determined using the pH drift method (M. Zbair, Z. Anfar, H. Khallok, H. Ait Ahsaine, M. Ezahri 2018; Zbair et al. 2018; Anfar et al. 2018). ANS@H2O-120 (0.20 g) was mixed with 50 mL of 0.01 M NaCl solution. The pH of the starting solutions (2.0 to 12.0) was adjusted using HCl and NaOH. After 24 h, the final pH was measured. The functional surface groups of ANS@H2O-30, ANS@H2O-90, and ANS@H2O-120 were determined using a Fourier transformed infrared spectroscopy (FTIR–8400S, Shimadzu).

**
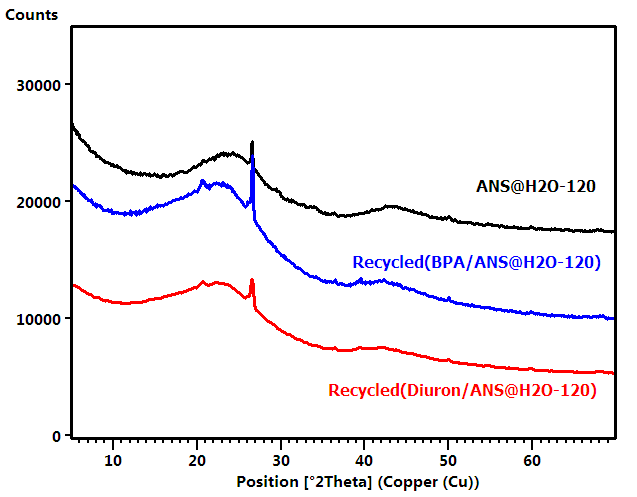
**

**Fig. 1S** XRD pattern of the fresh and the recycled ANS@H2O-120 (After 5 cycle of Adsorption).

**Table 1S** Equations used in this work to fit the data of adsorption experiments.

| **Equations** | **Utility** | **Description** | **References** |
| --- | --- | --- | --- |
| $Q_{e,t}=\frac{{(C}_{0}{-C}_{e,t}) \times V}{m}$ | adsorption capacity | C_0_ (mg/L) and C_e,t_ (mg/L) are the initial and equilibrium concentrations, respectively. m (g) is the weight of adsorbent and V (L) is the volume of BPA and diuron. | (Wang et al. 1998) |
| $Removal \%=\left( \frac{C_{0}{-C}_{e,t}}{C_{0}} \right) \times100$ | Removal efficiency | C_0_ (mg/L) and C_e,t_ (mg/L) are the initial and equilibrium concentrations of BPA and diuron. | (Garg et al. 2003) |
| $Q_{t}=Q_{\mathrm{cal}}(1-\exp^{K1t})$ | Pseudo-first-order | Q_e_ and Q_t_ are the adsorbed BPA and diuron amounts at equilibrium and at times t, respectively. K_1_: the rate constant. | (Lagergren 1898) |
| $Q_{t}=\frac{(K_{2}Q_{\mathrm{cal}}^{2}t)}{(1+K_{2}Q_{\mathrm{cal}}t)}$ | Pseudo-second-order | K_2:_ rate constant | (G.M. 1999) |
| $Q_{t}=K_{\mathrm{ip}}t^{\frac{1}{2}}+C$ | Intraparticle diffusion | Kip (mg/g.min-1/2) : rate coefficient; C: thickness of the boundary layer | (Weber, W.J. and Morris 1963) |
| $\frac{1}{Q_{e}}=\frac{1}{(K_{L}Q_{max})C_{e}}+\frac{1}{Q_{max}}$ | Langmuir model | C_e_ and Q_e_ are the concentration and amount at equilibrium; K_L_: direct measure of the intensity of the adsorption process; Q_max_: maximum adsorption capacity. | (Langmuir 1916) |
| $R_{L} = \frac{1}{1+ K_{L}C_{0}}$ | Adsorption feasability | The adsorption process can be de fined as irreversible (R_L_ = 0), favorable (R_L_ between 0 and 1), linear (RL = 1) or unfavorable (R_L_<1). | (Fan et al. 2017) |
| $lnQ_{e}= \frac{1}{n}\ln C_{e}+\ln K_{F}$ | Freundlich model | K_F_: adsorption capacity; n: intensity of adsorption; 1/n=0 irreversible; 1/n>1 unfavorable 0<1/n<1 favorable. | (Freundlich 1907) |
| ${\Delta G}^{^{\circ}}=-RTlnK_{d}$ | Gibbs free energy | ΔG°: Gibbs free energy change; Kd: equilibrium constant; R: gas constant; T: temperature. | (Tsai and Chang 1995) |
| $\mathrm{Ln}K_{d}=\frac{{\Delta S}^{^{\circ}}}{R}-\frac{{\Delta H}^{^{\circ}}}{RT}$ | Van’t Hoff | ΔS°: entropy change; ΔH°: enthalpy change. | (Hameed et al. 2007) |

**Table 2S** Independent parameters and response variables of matrix.

|  | | **Variable** | | **Name** | | **Unit** | **-1.68 (α)** | | **-1** | **0** | | **+1** | **+1.68 (α)** |
| --- | --- | --- | --- | --- | --- | --- | --- | --- | --- | --- | --- | --- | --- |
| **Bisphenol A** | | **X1** | | **pH** | | **-** | 2.2955 | | 4 | 6.5 | | 9 | 10.7045 |
|  |  | **X2** | | **Concentration** | | **mg/L** | 58.3182 | | 59 | 60 | | 61 | 61.6818 |
|  |  | **X3** | | **Contact time** | | **min** | 6.5911 | | 10 | 15 | | 20 | 23.4090 |
| **Diuron** | | **X1** | | **pH** | | **-** | 2.1660 | | 4 | 6.69 | | 9.38 | 11.2140 |
|  |  | **X2** | | **Concentration** | | **mg/L** | 38.3182 | | 39 | 40 | | 41 | 41.6818 |
|  |  | **X3** | | **Contact time** | | **min** | 6.5911 | | 10 | 15 | | 20 | 23.4090 |
| **Run no.** | **Operating parameters** | | | | | | | **BPA Removal %** | | | **Diuron Removal %** | | |
|  | **pH** | | **Concentration (mg/L)** | | **Contact time**  **(min)** | | |  |  |  |  |  |  |
| **1** | -1.00000 | | -1.00000 | | -1.00000 | | | 99.46 | | | 57.23 | | |
| **2** | 1.00000 | | -1.00000 | | -1.00000 | | | 73.05 | | | 99.61 | | |
| **3** | -1.00000 | | 1.00000 | | -1.00000 | | | 94.21 | | | 61.34 | | |
| **4** | 1.00000 | | 1.00000 | | -1.00000 | | | 75.04 | | | 99.72 | | |
| **5** | -1.00000 | | -1.00000 | | 1.00000 | | | 97.11 | | | 99.31 | | |
| **6** | 1.00000 | | -1.00000 | | 1.00000 | | | 73.08 | | | 99.65 | | |
| **7** | -1.00000 | | 1.00000 | | 1.00000 | | | 90.24 | | | 93.42 | | |
| **8** | 1.00000 | | 1.00000 | | 1.00000 | | | 89.44 | | | 99.54 | | |
| **9** | - α | | 0.00000 | | 0.00000 | | | 97.42 | | | 45.78 | | |
| **10** | α | | 0.00000 | | 0.00000 | | | 60.12 | | | 94.87 | | |
| **11** | 0.00000 | | - α | | 0.00000 | | | 92.91 | | | 99.91 | | |
| **12** | 0.00000 | | α | | 0.00000 | | | 96.91 | | | 98.90 | | |
| **13** | 0.00000 | | 0.00000 | | - α | | | 92.54 | | | 97.33 | | |
| **14** | 0.00000 | | 0.00000 | | α | | | 98.98 | | | 99.78 | | |
| **15** | 0.00000 | | 0.00000 | | 0.00000 | | | 99.53 | | | 96.07 | | |
| **16** | 0.00000 | | 0.00000 | | 0.00000 | | | 99.73 | | | 99.70 | | |
| **17** | 0.00000 | | 0.00000 | | 0.00000 | | | 96.98 | | | 99.88 | | |

**Table 3S** Analysis of variance and coefficients of determination.

|  |  | | | **Sum of squares** | **Degree**  **of freedom** | | | **Mean square** | **F-value** | **P-value** |
| --- | --- | --- | --- | --- | --- | --- | --- | --- | --- | --- |
| **Bisphenol A** | **Regression** | | | 2.19774E+0003 | 9 | | | 2.44193E+0002 | 20.7033 | < 0.0001 |
|  | **Residue** | | | 8.25641E+0001 | 7 | | | 1.17948E+0001 |  |  |
|  | **Lack-of-fit** | | | 7.78625E+0001 | 5 | | | 1.55725E+0001 | 6.6242 | 0.136>0.05 |
|  | **Error** | | | 4.70167E+0000 | 2 | | | 2.35083E+0000 |  |  |
|  | **Total** | | | 2.28030E+0003 | 16 | | |  |  |  |
|  | **R^2^** | | | 0.964 |  | | |  |  |  |
|  | **R^2^_Adj_** | | | 0.917 |  | | |  |  |  |
| **Diuron** |  | | | **Sum of squares** | **Degree**  **of freedom** | | | **Mean square** | **F-value** | **P-value** |
|  | **Regression** | | | 4.58332E+0003 | 9 | | | 5.09257E+0002 | 11.6029 | < 0.0001 |
|  | **Residue** | | | 3.07233E+0002 | 7 | | | 4.38904E+0001 |  |  |
|  | **Lack-of-fit** | | | 2.97991E+0002 | 5 | | | 5.95982E+0001 | 12.8975 | 0.074>0.05 |
|  | **Error** | | | 9.24180E+0000 | 2 | | | 4.62090E+0000 |  |  |
|  | **Total** | | | 4.89055E+0003 | 16 | | |  |  |  |
|  | **R^2^** | | | 0.937 |  | | |  |  |  |
|  | **R^2^_Adj_** | | | 0.856 |  | | |  |  |  |
| **Bisphenol A** | | **Nom** | **Coefficient** | | | **t.exp.** | **P-value** | | | |
|  |  | **a_0_** | 98.928 | | | 49.99 | < 0.0001 | | | |
|  |  | **a_pH_** | -9.749 | | | -10.49 | < 0.0001 | | | |
|  |  | **a_C_** | 0.949 | | | 1.02 | 0.341 | | | |
|  |  | **a_CT_** | 1.387 | | | 1.49 | 0.179 | | | |
|  |  | **a^2^_pH_** | -7.688 | | | -7.52 | 0.000135 | | | |
|  |  | **a^2^_C_** | -1.982 | | | -1.94 | 0.094 | | | |
|  |  | **a^2^_CT_** | -1.681 | | | -1.64 | 0.144 | | | |
|  |  | **a_pH-C_** | 3.809 | | | 3.14 | 0.0165 | | | |
|  |  | **a_pH-CT_** | 2.594 | | | 2.14 | 0.07 | | | |
|  |  | **a_C-CT_** | 1.594 | | | 1.31 | 0.231 | | | |
| **Diuron** | | **Nom** | **Coefficient** | | | **t.exp.** | **P-value** | | | |
|  |  | **a_0_** | 98.560 | | | 25.82 | < 0.0001 | | | |
|  |  | **a_pH_** | 12.432 | | | 6.93 | 0.000224 | | | |
|  |  | **a_C_** | -0.255 | | | -0.14 | 0.891 | | | |
|  |  | **a_CT_** | 5.722 | | | 3.19 | 0.0152 | | | |
|  |  | **a^2^_pH_** | -10.012 | | | -5.07 | 0.00144 | | | |
|  |  | **a^2^_C_** | 0.270 | | | 0.14 | 0.895 | | | |
|  |  | **a^2^_CT_** | -0.031 | | | -0.02 | 0.988 | | | |
|  |  | **a_pH-C_** | 0.223 | | | 0.09 | 0.927 | | | |
|  |  | **a_pH-CT_** | -9.287 | | | -3.97 | 0.00543 | | | |
|  |  | **a_C-CT_** | -1.277 | | | -0.55 | 0.602 | | | |

**References**

Anfar Z, Zbair M, Ahsaine HA, et al (2018) Well-designed WO3/Activated carbon composite for Rhodamine B Removal: Synthesis, characterization, and modeling using response surface methodology. Fullerenes, Nanotub Carbon Nanostructures 26:389–397. doi: 10.1080/1536383X.2018.1440386

Fan S, Wang Y, Wang Z, et al (2017) Removal of methylene blue from aqueous solution by sewage sludge-derived biochar: Adsorption kinetics, equilibrium, thermodynamics and mechanism. J Environ Chem Eng 5:601–611. doi: 10.1016/j.jece.2016.12.019

Freundlich H (1907) Über die Adsorption in Lösungen. Zeitschrift für Phys Chemie 57U: doi: 10.1515/zpch-1907-5723

G.M. M (1999) Pseudo-second order model for sorption process. Proc Biochem 34:451

Garg VK, Gupta R, Yadav AB, Kumar R (2003) Dye removal from aqueous solution by adsorption on treated sawdust. Bioresour Technol 89:121–124. doi: 10.1016/S0960-8524(03)00058-0

Hameed BH, Ahmad AA, Aziz N (2007) Isotherms, kinetics and thermodynamics of acid dye adsorption on activated palm ash. Chem Eng J 133:195–203. doi: 10.1016/j.cej.2007.01.032

Lagergren S (1898) Zur theorie der sogenannten adsorption gelöster stoffe [On the theory of so-called adsorption of dissolved substances]. Kungliga Svenska Vetenskapsakademiens. HandLingar 24:1

Langmuir I (1916) The constitution and fundamental properties of solids and liquids. Part I. Solids. J Am Chem Soc 38:2221–2295. doi: 10.1021/ja02268a002

M. Zbair, Z. Anfar, H. Khallok, H. Ait Ahsaine, M. Ezahri NE alem (2018) Adsorption kinetics and surface modeling of aqueous methylene blue onto activated carbonaceous wood sawdust. Fullerenes, Nanotub Carbon Nanostructures. doi: 10.1080/1536383X.2018.1447564

Tsai W, Chang C (1995) Surface characterization and thermodynamics of adsorption of methylene chloride on activated carbons. J Environ Sci Heal Part A Environ Sci Eng Toxicol 30:525–535. doi: 10.1080/10934529509376215

Wang J, Huang CP, Allen HE, et al (1998) Adsorption characteristics of dye onto sludge particulates. J Colloid Interface Sci 208:518–528. doi: 10.1006/jcis.1998.5875

Weber, W.J. and Morris JC (1963) Kinetics of adsorption carbon from solutions. J Sanit Engeering Div Proceedings Am Soc Civ Eng 89:31–60

Zbair M, Anfar Z, Ahsaine HA, et al (2018) Acridine orange adsorption by zinc oxide / almond shell activated carbon composite : Operational factors , mechanism and performance optimization using central composite design and surface modeling. J Environ Manage 206:383–397. doi: 10.1016/j.jenvman.2017.10.058
